# Irisin Exerts Inhibitory Effect on Adipogenesis Through Regulation of Wnt Signaling

**DOI:** 10.3389/fphys.2019.01085

**Published:** 2019-08-22

**Authors:** Eun Bi Ma, Namood E. Sahar, Moonsup Jeong, Joo Young Huh

**Affiliations:** ^1^College of Pharmacy, Chonnam National University, Gwangju, South Korea; ^2^GeneOne Life Science, Inc., Seoul, South Korea

**Keywords:** adipogenesis, FNDC5, irisin, Wnt, myokine

## Abstract

Irisin is an exercise-induced myokine known to induce adipocyte browning through induction of uncoupling protein 1. Recent studies have reported that irisin is also an adipokine. However, there is limiting evidence on the role of endogenous irisin from adipocytes. In this study we aim to elucidate the expression and secretion pattern of irisin during adipocyte differentiation and the role of endogenous and exogenous irisin on the adipogenic process. As such, recombinant irisin, plasmid expressing FNDC5 and small interfering RNA were utilized. Our results show that the gene expression of irisin precursor FNDC5 and irisin secretion increases at the early stage of adipogenesis. Both recombinant irisin treated cells and FNDC5-overexpressed cells resulted in inhibition of adipogenesis evidenced by downregulated C/EBPα, PPARγ, and FABP4 expression and reduced lipid accumulation. Further data showed that the inhibitory effect of irisin on adipogenesis is mediated though potentiation of Wnt expression, which is known to determine the fate of mesenchymal stem cells and regulate adipogenesis. Conversely, FNDC5 knockdown cells showed downregulated Wnt expression, but failed to further induce adipogenesis. This study suggests that both exogenous and endogenous irisin is able to inhibit adipogenesis and that activation of Wnt and subsequent repression of transcription factors is partly involved in this process. This provides a novel insight on the local effect of irisin on adipocytes and additional benefit to protect against obesity-related metabolic disorders.

## Introduction

Obesity is an epidemic rapidly increasing worldwide. Obesity leads to various medical problems such as type 2 diabetes, cardiovascular disease, and cancer (Keller and Lemberg, 2003; Wahba and Mak, 2007). The classical role of adipose tissue is to store energy but an imbalance between food intake and energy expenditure leads to accumulation of excess lipid in adipose tissue, causing dysregulation of adipocyte metabolism (Choe et al., 2016). Development of obesity involves two routes, the increase in the size of adipocytes (hypertrophy) or the increase in the number of adipocytes (hyperplasia) (Jo et al., 2009). The latter is achieved by increased adipogenesis, the process involving differentiation of preadipocytes into mature adipocytes. Adipogenesis is induced by a complex network of transcription factors, including peroxisome proliferator-activated receptor (PPARγ), CCAAT/enhancer binding proteins (such as C/EBPα), and fatty acid binding protein 4 (FABP4), which are under the control of epigenetic modifiers through the Wnt/β-catenin signaling (Ross et al., 2000; Rosen and MacDougald, 2006). PPARγ and C/EBPα are the master regulators involved in the early differentiation process of adipocytes. FABP4, on the other hand, is considered to be involved in mature adipocyte formation (Moseti et al., 2016). In contrast, the canonical Wnt/β-catenin signaling pathway, stimulated by ligands such as Wnt6, Wnt10a, and Wnt10b, has been shown to inhibit the early stages of adipogenic differentiation (Cawthorn et al., 2012). With this regard, regulation of Wnt signaling has been suggested as a therapeutic strategy to combat obesity and related metabolic disorders.

It is well-known that adipocytes possess endocrine function by secreting various factors, so-called adipokines (Kershaw and Flier, 2004). Adipose tissue enlargement causes adipokine dysfunction which implies an imbalance between pro-inflammatory and anti-inflammatory factors. The extension of adipose tissue increases secretion of pro-inflammatory factors such as IL-6, IL-8, IL-18, and TNFα (Makki et al., 2013). On the other hand, anti-inflammatory factors such as IL-10 and adiponectin are decreased (Fasshauer and Bluher, 2015). Dysregulation of adipokines results not only in development of insulin resistance and inflammation in local adipocyte environment but also affect the metabolism and function of other peripheral tissues thereby causing systemic metabolism impairment (Jung and Choi, 2014).

Exercise is an effective strategy to overcome obesity and metabolic diseases. In addition to the direct enhancement of muscle strength, exercise has many benefits on whole body metabolism, thereby protecting the body from the risk of metabolic disorder development (So et al., 2014; Schnyder and Handschin, 2015; Stanford and Goodyear, 2016). Interestingly, recent studies have suggested that similar to adipocytes, skeletal muscle can also secrete hormones, known as myokines. Myokines are induced through exercise, which suggests that myokines are responsible for the crosstalk between muscle and other tissues (Pedersen and Febbraio, 2012). Therefore, myokines are suggested to counteract the increased pro-inflammatory adipokines during obesity (Leal et al., 2018). The exercise-induced myokines discovered so far include irisin, IL-15, SPARC, BDNF, FGF-21, etc. (Huh, 2018). These myokines are reported to modulate glucose uptake and improve glucose tolerance, regulate fat oxidation, and reduce fat accumulation in adipocytes (So et al., 2014). Among these, irisin is a myokine dependent on activation of peroxisome proliferator-activated receptor gamma coactivator 1-alpha (PGC1α), which is a major mediator of the effect of exercise in muscle (Lukaszuk et al., 2015). It was found that PGC1α overexpression leads to increased expression of fibronectin type III domain-containing protein 5 (FNDC5), which is cleaved to produce irisin and then secreted by exercise (Bostrom et al., 2012; Gouveia et al., 2016). The major role of secreted irisin is suggested to be browning of adipocytes through upregulation of uncoupling 1 (UCP1) expression (Bostrom et al., 2012), which has advantages in increasing energy expenditure and thus leads to improvement in metabolic health (Bartelt and Heeren, 2014; Panati et al., 2016). In addition, numerous studies have reported diverse action of irisin on the metabolism. Irisin is reported to increase mitochondrial content and function in cultured myocytes (Vaughan et al., 2014). Also, irisin treatment on human adipocytes inhibited lipid accumulation by increasing adipose triglyceride lipase (ATGL) and decreasing fatty acid synthase (FAS) (Huh et al., 2014).

Recent studies have suggested that irisin is not only a myokine but also an adipokine (Moreno-Navarrete et al., 2013; Roca-Rivada et al., 2013). Albeit low levels compared to muscle, expression and secretion of irisin/FNDC5 was confirmed in both mouse and human adipose tissue and cultured adipocytes. However, little is known about the functional role of adipocyte-derived irisin on metabolism. In this study, we investigated how irisin is regulated during adipocyte differentiation and then the role of endogenous and exogenous irisin in regulation of adipocyte differentiation was elucidated. Our results show that irisin and its precursor FNDC5 are expressed at early stages of adipogenesis and that ectopic expression of FNDC5 inhibits adipocyte differentiation. Moreover, we revealed that exogenous irisin led to activation of Wnt ligand expression. These findings suggest that irisin/FNDC5 disturbs adipogenic differentiation partially through regulation of Wnt signaling pathway.

## Materials and Methods

### Cell Culture

3T3-L1 mouse preadipocytes were purchased from ATCC (Manassas, VA, United States). For maintenance of preadipocytes, 100 mm culture plates were used. For gene expression analysis and Western blotting the cells were seeded in 6 well plates, whereas 96 well plates were used for cell viability assay and Oil Red O staining. The cells were maintained in Dulbecco modified Eagle medium (DMEM) with 10% FBS (HyClone, Australia) and 1% penicillin/streptomycin (Carlsbad, CA, United States) and incubated at 37°C in 5% CO_2_. 3T3-L1 cells were treated with differentiation media (MDI; methylisobutylxanthine, dexamethasone, insulin) to induce adipogenesis as previously described (Huh et al., 2012). To examine the effect of irisin on adipogenesis, 100 ng/mL recombinant irisin was treated every other day for 6 days, starting from 2 days before changing to MDI. Recombinant irisin was purchased from Phoenix Pharmaceuticals, Inc. (Burlingame, CA, United States). Plasmid encoding FNDC5 gene and small interfering RNA were also used to examine the effect of genetic manipulation of irisin on adipocyte differentiation, as described below.

### Cell Viability Assay

Cell viability was examined using 3-(4,5-dimethylthiazol-2-yl)-2,5- diphenyl-tetrazolium-bromide (MTT) assay reagent. When the 3T3-L1 preadipocytes reached 50% confluence, recombinant irisin was treated in various concentrations for 24 and 48 h. Then MTT assay reagent was added to a final concentration of 0.5 mg/mL. The cells were then incubated for 2 h until the purple formazan crystals are displayed under the microscope. Then, DMSO was added and incubated for 10 min before measuring the absorbance at 540 nm.

### Gene Expression Analysis

Total RNA was extracted from 3T3-L1 using TRI Reagent (MRC, Cincinnati, OH, United States). cDNA was synthesized using TOPscript^TM^ RT DryMIX (Enzynomics, South Korea). mRNA levels were measured by real-time PCR using Rotor-Gene Q (QIAGEN) with 20 μL reaction volume consisting of cDNA transcripts, primer pairs, and TOPreal SYBR Green PCR Kit (Enzynomics, South Korea). The gene expressions were normalized to 18S rRNA levels.

### Western Blotting

Cells were harvested and lysed with RIPA buffer (Thermo Scientific, Rockford, IL, United States). Prepared protein sample were separated by SDS-PAGE and transferred to PVDF membrane. Primary antibodies were incubated at 4°C overnight. After overnight incubation, secondary antibody was incubated at room temperature for 1 h and the blots were detected with LAS-4000 (Fuji Photo Film, Tokyo, Japan). PPARγ (#2435), C/EBPα (#8178), and FABP4 (#3544) antibodies were purchased from Cell Signaling Technology (Danvers, MA, United States). Wnt10a antibody (#sc-376028) was obtained from Santa Cruz Biotechnology (Santa Cruz, CA, United States). FNDC5 antibody (#SAB1301655) was obtained from Sigma-Aldrich (St. Louis, MO, United States). β-tubulin antibody (#MA5-16308) was obtained from Thermo Scientific (Rockford, IL, United States).

### Enzyme-Linked Immunosorbent Assay

Irisin secretion in the culture medium of adipocytes was measured with commercially available ELISA kits (Phoenix Pharmaceuticals, Burlingame, CA, United States), according to the manufacturer’s instruction. Culture medium was harvested every 2 days starting from day 0 of adipocyte differentiation.

### Cell Transfection

For the expression of irisin precursor FNDC5 gene, a plasmid expressing FNDC5 was generated using a modified pVAX1 mammalian expression vector. The gene encoding murine FNDC5 were genetically optimized for enhanced expression, including codon and RNA optimization, and a highly efficient immunoglobulin E leader sequence was added to facilitate expression. The construct was synthesized commercially, sequence verified, and then the optimized gene was subcloned into a modified pVAX1 vector under control of the cytomegalovirus immediate-early promoter. Sequence analysis was done by using GeneArt program from Life Technologies (Supplementary file). The DNA plasmid was designated as pVAX1-mFNDC5. 3T3-L1 cells were transfected with pVAX1-mFNDC5. Turbofect transfection reagent (Thermo Scientific, United States) and 1 μg of plasmid DNA were treated when the cells reached 80% confluence (4 days before adipocyte differentiation). Four days after transfection, transfected cells were differentiated according to the differentiation protocol. A modified pVAX1 mammalian expression vector was transfected as a negative control.

Commercially available FNDC5 small-interfering RNA (siRNA) and non-targeting siRNA as a negative control (Bioneer, South Korea) were used at 25 nmol/L. FNDC5 siRNA sequence is as follows: sense CAAGGUGCACCUUUGCAAA (dTdT), antisense UUUGCAAAGGUGCACCUUG (dTdT). Opti-MEM transfection media and lipofectamine (both from Invitrogen, Paisley, United Kingdom) were used for transfection, following the manufacturer’s protocol. Transfection was performed on 3T3-L1 2 days before induction of adipogenesis. One day before transfection, the growth media was replaced with 10% FBS media without penicillin/streptomycin. Cells were treated with silencing reagent/siRNA mixture for 48 h and were harvested to examine the transfection efficiency. For the effect of silencing on Wnt expression, the cells were harvested 1 day after MDI treatment.

### Oil Red O Staining

Oil Red O (ORO) staining was performed 8 days after induction of differentiation. Briefly, the cells were fixed with 10% formalin for 1 h. Then the cells were stained with 0.3% ORO solution (Sigma-Aldrich, St. Louis, MO, United States) for 2 h. After staining, the cells were washed with distilled water and representative pictures were taken under the microscope. For quantification, 100% isopropanol was added and the absorbance was measured at 490 nm.

### Statistical Analysis

All statistical analysis was performed using Statview software. Mean values obtained from three independent experiments were compared by ANOVA with subsequent Fisher’s significant difference method for *post hoc* paired comparisons. *P*-value of < 0.05 was used as the criterion for a statistically significant difference.

## Results

### Changes in FNDC5 Gene Expression and Irisin Secretion During Adipocyte Differentiation

**FIGURE 1:**
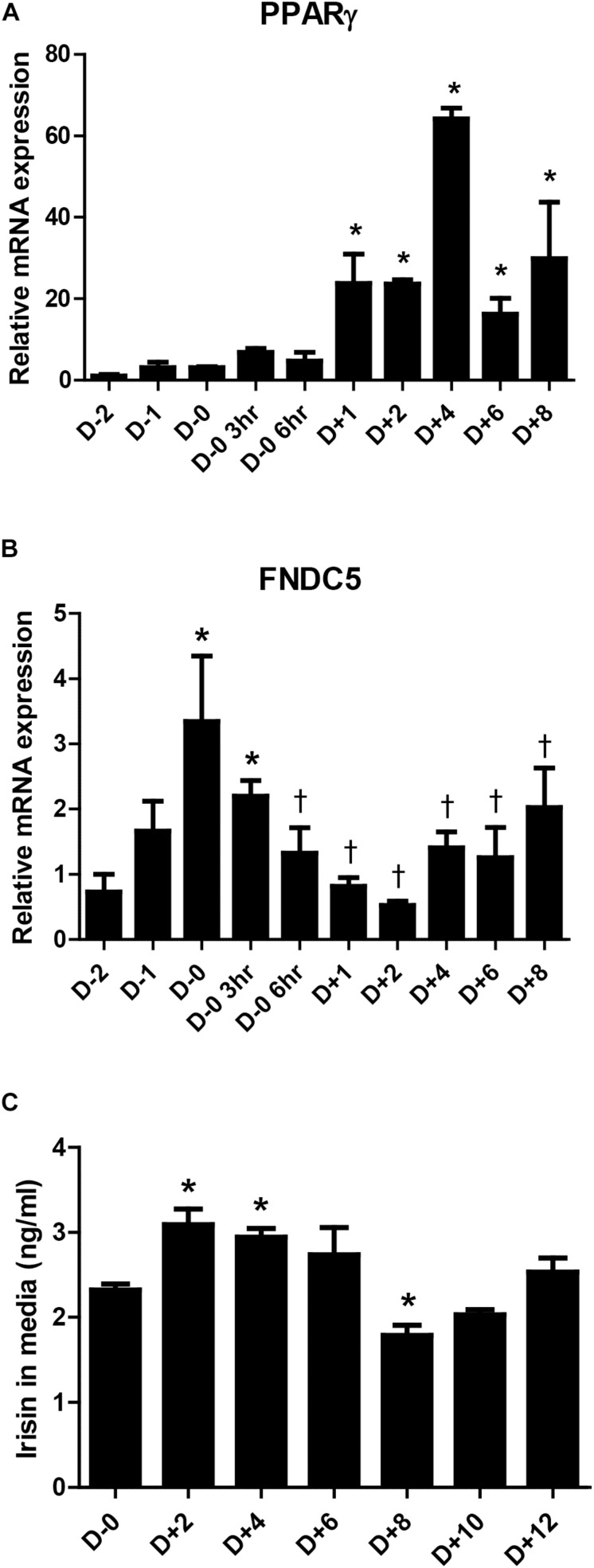
Irisin gene expression and secretion during 3T3-L1 adipogenesis. **(A,B)** Gene expressions of PPARγ and FNDC5 were examined by real-time PCR during day D–2 to D+8 of adipogenesis. Quantifications were normalized to 18S in each reaction. ^∗^*P* < 0.05 compared with D–2, †*P* < 0.05 compared with D–0. **(C)** Irisin secretion in media during adipogenesis was analyzed by ELISA. ^∗^*P* < 0.05 compared with D–0.

Previous studies have suggested that irisin is not only a myokine but also an adipokine (Moreno-Navarrete et al., 2013; Roca-Rivada et al., 2013; Huh et al., 2014). However, there is limiting evidence on the changes in its expression during adipogenesis. As such, the gene expression of FNDC5 during adipocyte differentiation was examined. The significant increase in PPARγ mRNA expression between days 1 to 8 during 3T3-L1 differentiation is shown as a positive marker of adipogenesis (Figure 1A). Concurrently, the gene expression of FNDC5 was the highest at day 0 (before induction of adipocyte differentiation) and significantly decreased after adipogenic stimulation by MDI treatment (Figure 1B), where the level was lowest on day 2 and gradually increased thereafter. This implies that the adipogenic cocktail is a strong stimulant for downregulation of FNDC5 gene expression. Next, the secretion of irisin during adipocyte differentiation was examined by performing ELISA in the conditioned medium. As a result, significantly increased secretion of irisin was observed between days 2 and 4 of adipocyte differentiation (Figure 1C), which is later period than increased expression of FNDC5 gene. Similar to the pattern of mRNA expression, secretion of irisin was highest at early stage (day 2) and then gradually decreased until day 8. These results confirm that irisin is an adipokine, where its mRNA expression and secretion are increased at early stage of adipocyte differentiation.

### Endogenous and Exogenous Irisin Both Induce Suppression of Adipocyte Differentiation

**FIGURE 2:**
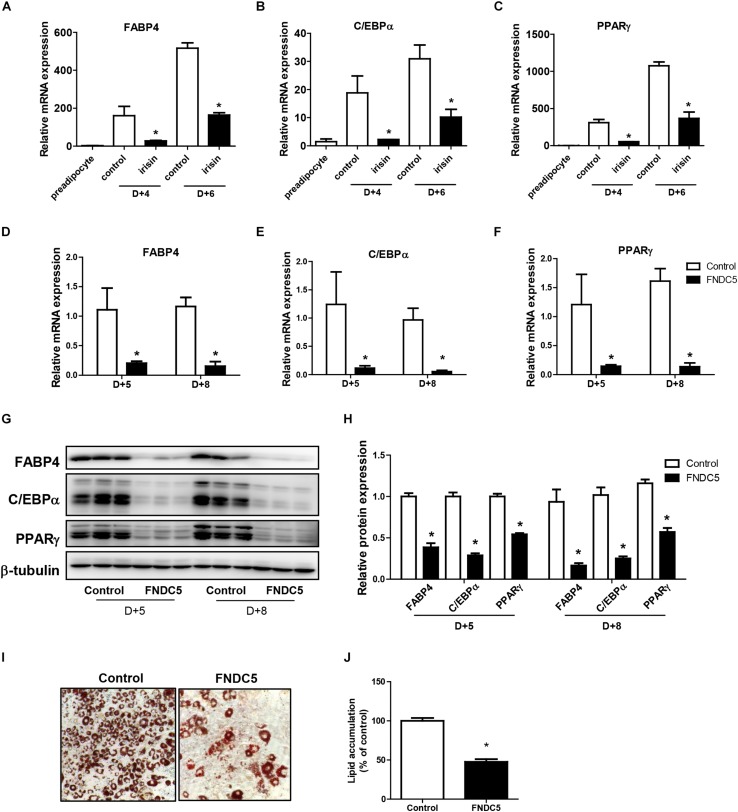
Effect of recombinant irisin and FNDC5 overexpression on adipogenesis. **(A–C)** 3T3-L1 cells were treated with or without 100 ng/ml irisin from D–2 to D+6 of adipogenesis. Gene expressions of transcription factors were measured by real-time PCR and normalized to 18S rRNA. **(D–J)** 3T3-L1 preadipocytes were transfected with control or plasmid expressing FNDC5. Two days confluent cells were induced to differentiate and were harvested at day + 5 and day + 8. **(D–F)** Gene expressions of PPARγ, CEBPα, and FABP4 were analyzed by real-time PCR and normalized to 18S. **(G,H)** Western blot analysis of transcription factors. **(I,J)** Representative pictures and quantification of Oil red O staining in FNDC5-overexpressed cells at day 8. ^∗^*P* < 0.05 compared with control at each time-point.

First, the effect of recombinant irisin on cell viability was tested. Preadipocytes were treated with recombinant irisin at concentrations 50, 100, and 200 ng/ml for 24 and 48 h. The results showed that recombinant irisin does not exert any toxic effect on preadipocytes (Supplementary Figure S1). As previously reported, FABP4, C/EBPα, and PPARγ are the key regulators of adipogenesis responsible for the formation of mature adipocytes (Moseti et al., 2016). To evaluate the role of exogenous irisin on adipocyte differentiation, adipocytes were treated with 100 ng/mL recombinant irisin during adipogenesis. Gene expressions of FABP4, C/EBPα, and PPARγ were increased in time-dependent manner at day 4 and day 6 of differentiation period, whereas irisin treatment significantly downregulated the expression of these transcription factors at both days (Figures 2A–C).

To further validate the role of endogenous irisin on adipocyte differentiation, FNDC5 was overexpressed in 3T3-L1 preadipocytes. Transfection efficiency was confirmed by the significantly increased FNDC5 mRNA expression compared with control (Supplementary Figure S2A). Also, protein levels were assessed by using anti-FNDC5 antibody. Similar to the pattern shown in previous findings (Perez-Sotelo et al., 2017; Zhang et al., 2017), both bands corresponding to the full-length FNDC5 protein and the cleaved irisin peptide were detected (Supplementary Figure S2C). Interestingly, while both FNDC5 and irisin were increased in FNDC5-overexpressed preadipocytes, only the increase in irisin levels was statistically significant (Supplementary Figure S2D). In line with the results from recombinant irisin treatment, gene expressions of FABP4, C/EBPα, and PPARγ were significantly lower in FNDC5-overexpressed cells compared with control at day 5 and day 8 (Figures 2D–F). Consistently, protein levels of FABP4, C/EBPα, and PPARγ were also significantly lower in FNDC5-overexpressed cells (Figures 2G,H). Moreover, Oil red O staining performed at day 8 showed a significant decrease in the number of mature adipocytes in FNDC5-overexpressed cells compared to control, which resulted in less accumulation of lipids (Figures 2I,J). These results imply that irisin endogenously produced from adipocytes possesses the ability to inhibit adipocyte differentiation through regulation of transcription factors, corresponding to the effect of recombinant irisin treatment.

### Wnt Expression Is Induced by Recombinant Irisin Treatment During Adipocyte Differentiation

**FIGURE 3:**
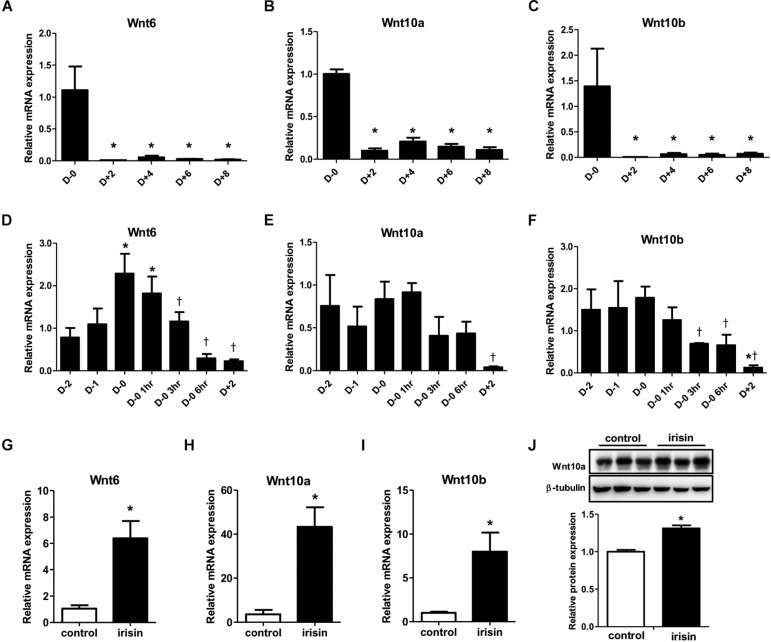
Effect of recombinant irisin treatment on Wnt expression. **(A–C)** Gene expression analysis of Wnt ligands during 3T3-L1 adipocyte differentiation by real-time PCR. Quantification were normalized to 18S rRNA ^∗^*P* < 0.05 compared with D–0. **(D–F)** Gene expression of analysis of Wnt ligands at early stage of adipogenesis by real-time PCR. ^∗^*P* < 0.05 compared with D–2, †*P* < 0.05 compared with D–0. **(G–J)** 3T3-L1 cells were treated with 100 ng/ml recombinant irisin at D–2. **(G–I)** Wnt gene expressions were measured by real-time PCR and normalized to 18S rRNA. **(J)** Protein expression of Wnt10a with or without recombinant irisin treatment were analyzed by Western blot, ^∗^*P* < 0.05 compared with control.

Previous studies have shown that Wnt signaling influences the fate of many cell types and cell differentiation (Logan and Nusse, 2004; Rosen and MacDougald, 2006). Especially, Wnt signaling is reported to be involved in inhibition of adipocyte differentiation (Rosen and MacDougald, 2006). Since numerous reports have shown varying patterns in Wnt expression during adipogenesis (Shen et al., 2011; Cawthorn et al., 2012), we first monitored the gene expressions of various Wnt ligands during adipocyte differentiation. The gene expressions of Wnt6, 10a, and 10b were markedly reduced from day 2 compared with day 0 (Figures 3A–C). To gain further insight into the pattern of Wnt decrease, changes in the gene expression from 2 days before initiation of adipogenesis (D−2) until day 2 was analyzed in detail. The expression of Wnt6 was highest at day 0 and started to decrease from 3 h after changing the media to adipogenic cocktail (Figure 3D), which is similar to the mRNA expression pattern of FNDC5. Wnt10a and Wnt10b had similar pattern where the expression level was stable until day 0, and then the expression decreased from 3 h after MDI treatment (Figures 3E,F). Taken together, it was confirmed that Wnt expression is dramatically reduced by adipogenic stimulation.

Subsequent to the previous results on the effect of irisin on transcription factors, we investigated whether the inhibitory effect of irisin on adipogenesis is associated with regulation of Wnt. In 3T3-L1 preadipocytes, recombinant irisin was treated from 2 days before initiation of differentiation (D−2). Interestingly, the gene expressions of Wnt6, 10a, and 10b on day 2 of adipogenesis were significantly restored by irisin treatment compared to untreated control (Figures 3G–I). The protein expression of Wnt10a was also significantly increased by recombinant irisin treatment (Figure 3J), although to a lesser extent than observed with the change in gene expression. In summary, above results imply that irisin induces the expression of Wnt, which would subsequently disrupt adipogenesis through controlling transcription factors.

### Effect of FNDC5 Silencing on Adipogenesis

**FIGURE 4:**
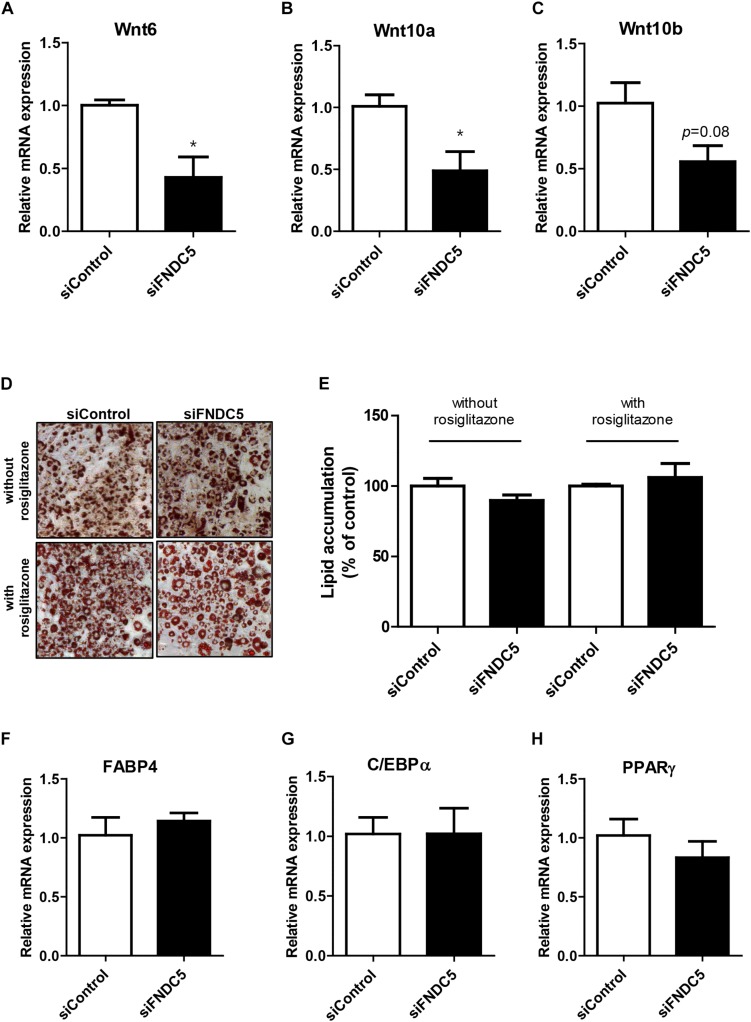
Effect of FNDC5 silencing on Wnt gene expression and adipogenesis. 3T3-L1 preadipocytes were transfected with FNDC5 siRNA and negative control at 25 nmol/L for 48 h. **(A–C)** Cells were harvested 1 day after MDI treatment. Gene expression of Wnt ligands were examined by real-time PCR and normalized to 18S in each reaction. **(D,E)** Representative pictures and quantification of Oil red O staining after siFNDC5 transfection. The adipogenesis was induced by conventional MDI treatment or with addition of PPARγ agonist, rosiglitazone, and harvested on day 8. **(F–H)** Real-time PCR analysis of transcription factors after siFNDC5 transfection. Data is shown as ^∗^*P* < 0.05 compared with siControl.

Next, we sought to examine whether reduction in endogenous irisin expression would lead to the opposite effect. To verify, gene silencing was conducted using FNDC5 siRNAs in 3T3-L1 cells. Significant decrease in FNDC5 gene expression confirmed the transfection efficiency of siRNA (Supplementary Figure S2B). As expected, gene expressions of Wnt6 and Wnt10a were significantly decreased in FNDC5 knockdown (FNDC5-KD) cells compared to preadipocytes treated with negative siRNA (Figures 4A,B). Wnt10b gene expression was also marginally decreased (Figure 4C).

Since alteration in endogenous FNDC5 level led to changes in Wnt expression, we further evaluated whether FNDC5 silencing leads to regulation of adipogenesis. In contrast to the effects observed in FNDC5 overexpressed cells, Oil red O staining performed at day 8 of adipogenesis showed no difference in the extent of adipocyte differentiation between FNDC5-KD and control cells as evidenced by similar levels of lipid accumulation (Figures 4D,E). The results were similar whether the cells were differentiated using conventional MDI or with addition of PPARγ agonist rosiglitazone. In line with this observation, the gene expressions of adipogenic transcription factors were also unaltered in response of FNDC5 silencing (Figures 4F–H). These data suggest that although Wnt expression is partially affected by inhibition of FNDC5 levels, it was not sufficient to regulate the differentiation of adipocytes.

## Discussion

**FIGURE 5:**
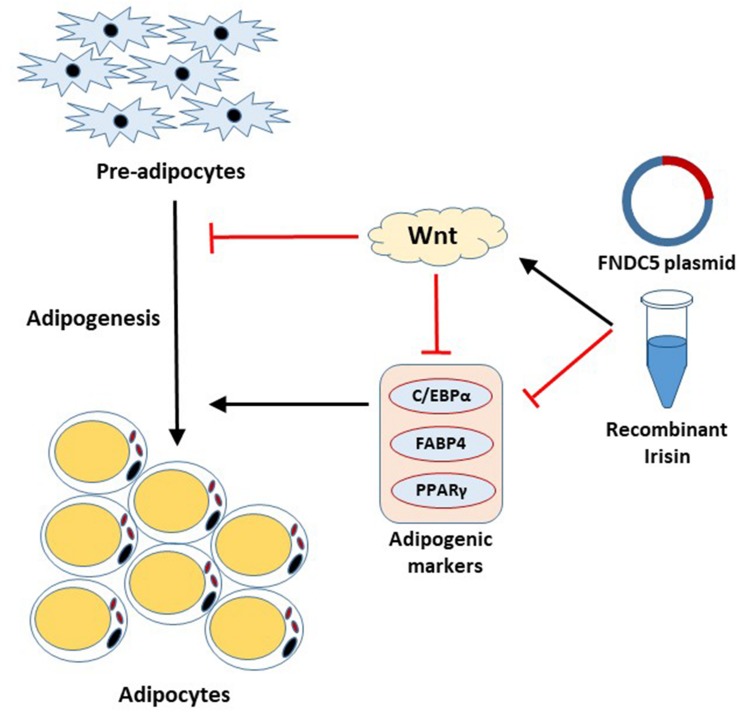
Schematic diagram of the effect of irisin on adipogenesis.

Irisin has received a significant amount of attention since its discovery as adipocyte browning factor (Bostrom et al., 2012). Recent findings show pluripotent role of this exercise-induced hormone in various tissues, which emphasizes the beneficial role of exercise on whole body metabolism (Perakakis et al., 2017; Askari et al., 2018; de Oliveira Bristot et al., 2019; Farmer, 2019). The present study unveiled the significant role of FNDC5/irisin on adipogenesis. This study, for the first time, reports that irisin exhibits inhibitory effects on adipogenesis through regulation of Wnt signaling. The important aspects of our findings are; (1) FNDC5 mRNA expression and irisin secretion are increased at the early stage of adipogenesis, (2) Endogenous as well as exogenous irisin regulates the adipocyte differentiation through downregulation of the transcription factors, (3) Irisin inhibits adipocyte differentiation by inducing Wnt expression, (4) FNDC5 silencing in adipocytes alters the Wnt expression but shows no significant effects on adipocyte differentiation and lipid accumulation. These results provide evidence on the intrinsic role of irisin in adipocytes (Figure 5).

Previous studies have shown the potential role of exercise-induced irisin in the treatment of various diseases. These reports have highlighted the role of irisin as a myokine, mediating the beneficial effects of exercise on adipose tissue, liver, bone, brain, etc. through endocrine effect (Huh et al., 2014; Zhang et al., 2017; Bi et al., 2019; Lourenco et al., 2019). Although muscle is responsible for the majority of secreted irisin in circulation, reports are continuously appearing suggesting the local expression of irisin in other organs (Colaianni et al., 2015; Wahab et al., 2019). Similar to other myokines reported to date, irisin is also reported as an adipokine (Moreno-Navarrete et al., 2013; Roca-Rivada et al., 2013; Huh et al., 2014; Zhang et al., 2016; Perez-Sotelo et al., 2017). It is interesting to note that in the case of obesity, irisin seems to be over-secreted from adipose tissue which may be an adaptive response to counterbalance metabolic dysregulation (Moreno-Navarrete et al., 2013; Roca-Rivada et al., 2013). However, the functional role of adipocyte-derived irisin has not been studied in detail. We and others have previously reported that in addition to the role of irisin in inducing adipocyte browning, irisin has the ability to inhibit adipocyte differentiation (Huh et al., 2014; Zhang et al., 2016). In the current study, we found that FNDC5-overexpressed preadipocytes are inhibited from adipogenic differentiation, suggesting the intrinsic role of adipocyte-derived irisin. Interestingly, both results from recombinant irisin treatment and FNDC5 overexpression show that there are significant inhibitory effects during the differentiation process (days 4–5) and after the adipogenesis is completed (days 6–8). ORO staining at day 8 shows a contrast in number of mature adipocytes compared to control. We further found that the inhibitory effect of irisin on adipogenesis is mediated via activation of Wnt, which is known to repress adipogenesis by blocking the induction of PPARγ and C/EBPα, as shown in our results (Kennell and MacDougald, 2005; Cawthorn et al., 2012). It remains to be studied whether downstream signaling of Wnt such as β-catenin is also involved in this process. Furthermore, there is immense need to discuss the effect of Wnt agonist and inhibitors on the anti-obesity and anti-diabetic effects of irisin.

Canonical Wnt signaling is known to control the balance between myogenesis, osteogenesis, and adipogenesis (Christodoulides et al., 2009; Cawthorn et al., 2012). Accordingly, it has been recently reported by several groups that irisin regulates bone metabolism through increasing osteoblastogenesis and mineralization (Colaianni et al., 2015; Zhang et al., 2017). Not surprisingly, this effect was mediated through downregulation of sclerotin, a Wnt/β-catenin pathway inhibitor. Therefore, the activation of Wnt by irisin seems to exert dual effects by enhancing bone strength while limiting the expansion of adipose tissue through inhibition of adipocyte formation. Similarly, other myokines are also reported to regulate the Wnt signaling. For example, myostatin is known to inhibit osteoblastic differentiation (Qin et al., 2017) and SPARC is known to inhibit adipocyte differentiation and adipose tissue turnover (Nie and Sage, 2009) through regulation of Wnt pathway, which suggest a common role of exercise-induced myokines.

In accordance with our data on the effect of ectopic expression of FNDC5, a recent study has reported that adipocytes lacking FNDC5 gene expression show increased accumulation of lipids during adipogenesis (Perez-Sotelo et al., 2017). In our study, although we have observed significantly reduced gene expression of Wnt ligands by knockdown of FNDC5, the rate of adipocyte differentiation did not differ between FNDC5-KD and control cells. In the study by Perez-Sotelo et al. (2017), FNDC5 was knocked down using shRNA with lentiviral infection which resulted in over 90% reduction in gene expression, whereas in our data the reduction rate was around 70–80%. In addition, the discrepancy may derive from the difference in the cell lines used (3T3-L1 versus C3H10T1/2 murine mesenchymal stem cells). This idea is supported by the fact that the two studies show difference in the expression profile of FNDC5 during adipocyte differentiation. Perez-Sotelo et al. (2017), showed a gradual increase in FNDC5 expression from day 2 to 10, paralleling the expression of PPARγ. In contrast we observed the increase in both FNDC5 gene expression and irisin secretion at the early stage of adipogenesis. This is in line with our previous report where the FNDC5 mRNA level in mature adipocytes was significantly lower than stromal vascular cells in humans (Huh et al., 2014). Of note, the mRNA expression pattern of FNDC5 during adipogenesis showed similarity to that of Wnt6, providing a plausible relationship between the two factors that negatively regulate adipogenesis. It is also possible that since the basal FNDC5 levels in preadipocytes are very low [several 100-fold lower compared to myocytes (Moreno-Navarrete et al., 2013; Huh et al., 2014)], silencing has minor influence on the cells. Further studies are needed at *in vitro* and *in vivo* level to resolve the controversy.

Irisin is produced as a result of cleavage of its precursor, FNDC5 (Bostrom et al., 2012) Studies have shown that increase in FNDC5 is directly involved with elevated circulating irisin levels (Xiong et al., 2015; Geng et al., 2019). In our study, we have shown that similar outcome is brought by recombinant irisin treatment and induction of endogenous FNDC5 expression. This partly suggests that regulation of FNDC5 expression leads to changes in irisin secretion. It is important to note that the protease responsible for cleavage of FNDC5 is currently unknown. Therefore, it is possible that irisin secretion could be induced by induction of FNDC5 cleavage not expression *per se*. Following secretion, irisin is expected to exert autocrine effect through recently identified irisin receptor aV integrin (Kim et al., 2018). This receptor is reported to exist in fat and is responsible for the effect of irisin in inducing thermogenic genes. Whether this receptor is also responsible for the effect of irisin on inhibition of adipogenesis should further be studied in the future.

In conclusion, we found that FNDC5/irisin levels are induced at early process of adipocyte differentiation, and this leads to inhibitory effect on adipogenesis through regulation of Wnt. Moreover, we have demonstrated that endogenous expression of FNDC5/irisin is able to act on adipocytes in an autocrine/paracrine manner. In addition to the browning effect of irisin on mature adipocytes, our data provide evidence on its ability to limit expansion of adipose tissue, which further potentiates its therapeutic potential in obesity-related metabolic disorders.

## Data Availability

All datasets generated for this study are included in the manuscript and/or the Supplementary Files.

## Author Contributions

EM, NS, MJ, and JH conceived and designed the study, and drafted and critically revised the manuscript. EM, NS, and JH performed the experiments and analyzed the data. All authors approved the final version of the manuscript.

## Conflict of Interest Statement

MJ was employed by company GeneOne Life Science, Inc. The remaining authors declare that the research was conducted in the absence of any commercial or financial relationships that could be construed as a potential conflict of interest.
