# Supplementary material for: Irisin Exerts Inhibitory Effect on Adipogenesis Through Regulation of Wnt Signaling
Source: Front Physiol. 2019 Aug 22;10:1085. doi: 10.3389/fphys.2019.01085 (PMC6714492; doi:10.3389/fphys.2019.01085)
Supplement: Supplementary file 1 [file Data_Sheet_1.PDF]

Supplementary Material

1 Supplementary Data

Sequence analysis for plasmid expressing FNDC5

1.1 Methods

The codon usage was adapted to the codon bias of *Mus musculus* genes. In addition, regions of very high (> 80 %) or very low (< 30 %) GC content have been avoided where possible.

During the optimization process the following cis-acting sequence motifs were avoided where applicable; internal TATA-boxes, chi-sites and ribosomal entry sites, AT-rich or GC-rich sequence stretches, RNA instability motifs, repeat sequences and RNA secondary structures, (cryptic) splice donor and acceptor sites in higher eucaryotes

1.2 Results

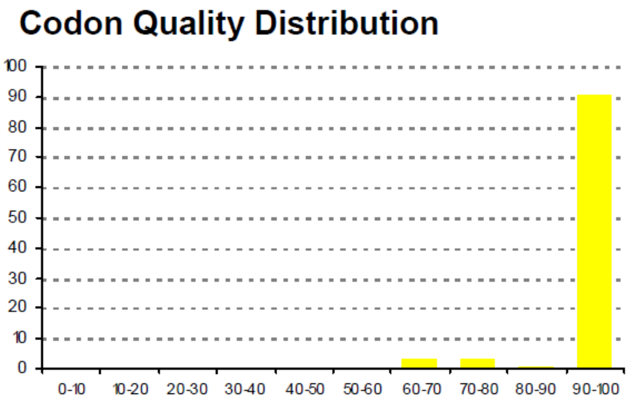

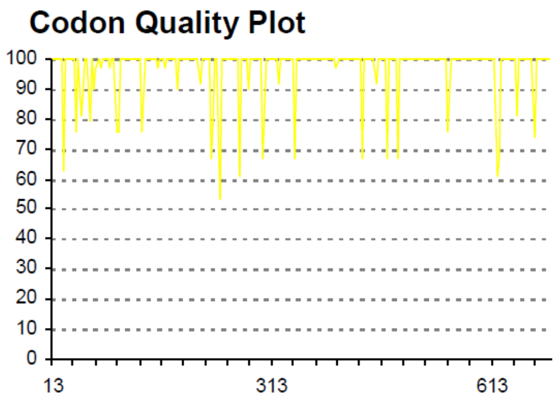

Codon Adaptation Index : 0.96

The histograms show the percentage of sequence codons which fall into a certain quality class. The quality value of the most frequently used codon for a given amino acid in the desired expression system is set to 100, the remaining codons are scaled accordingly. The plots show the quality of the used codon at the indicated codon position.

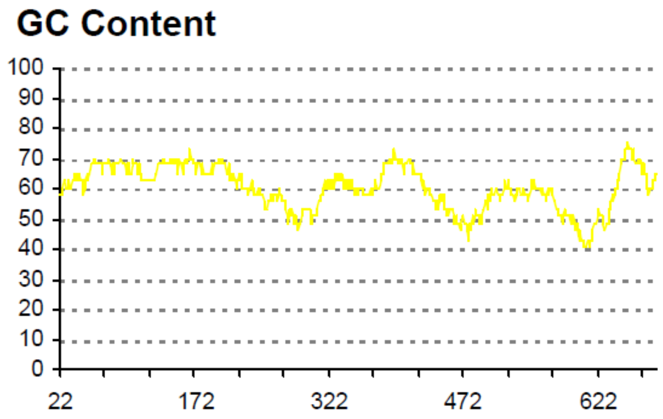

Average GC content: 60 %

The plot shows the GC content in a 40 bp window centered at the indicated nucleotide position.

1.3 Summary

The optimization was successful: Negative cis-acting sites (such as splice sites, TATA-boxes, etc.) which may negatively influence expression were eliminated wherever possible. GC content was adjusted to prolong mRNA half-life. Codon usage was adapted to the bias of *Mus musculus* resulting in a CAI* value of 0.96. The optimized gene should therefore allow high and stable expression rates in *Mus musculus*.

* CAI: The parameter CAI (codon adaptation index) describes how well the codons match the codon usage preference of the target organism. Thus, a CAI of 1.0 would be perfect. However, a CAI of > 0.9 is considered as very good (i.e. allowing high expression).

** In some rare cases negatively cis acting motifs cannot be removed due to the particular amino acid sequence or restriction site

1.4 Optimized sequence

```
                 PflMI
     BamHI       NcoI
     GGATCCGCCACCATGGACTGGACCTGGATTCTGTTCCTGGTGGCCGCTGCCACACGGGTG
   1 ---------+---------+---------+---------+---------+---------+
     CCTAGGCGGTGGTACCTGACCTGGACCTAAGACAAGGACCACCGGCGACGGTGTGCCCAC
                 M__D__W__T__W__I__L__F__L__V__A__A__A__T__R__V__
                 1     3     5     7     9     11    13    15

     CACTCTATGCCACCTGGCCCTTGCGCCTGGCCTCCCAGAGCTGCTCTGAGACTGTGGCTG
  61 ---------+---------+---------+---------+---------+---------+
     GTGAGATACGGTGGACCGGGAACGCGGACCGGAGGGTCTCGACGAGACTCTGACACCGAC
     H__S__M__P__P__G__P__C__A__W__P__P__R__A__A__L__R__L__W__L__
     17    19    21    23    25    27    29    31    33    35

     GGCTGCGTGTGCTTCGCTCTGGTGCAGGCCGACAGCCCTAGCGCCCCTGTGAACGTGACC
 121 ---------+---------+---------+---------+---------+---------+
     CCGACGCACACGAAGCGAGACCACGTCCGGCTGTCGGGATCGCGGGGACACTTGCACTGG
     G__C__V__C__F__A__L__V__Q__A__D__S__P__S__A__P__V__N__V__T__
     37    39    41    43    45    47    49    51    53    55

     GTGCGGCACCTGAAGGCCAACAGCGCCGTGGTGTCCTGGGACGTGCTGGAAGATGAGGTG
 181 ---------+---------+---------+---------+---------+---------+
     CACGCCGTGGACTTCCGGTTGTCGCGGCACCACAGGACCCTGCACGACCTTCTACTCCAC
     V__R__H__L__K__A__N__S__A__V__V__S__W__D__V__L__E__D__E__V__
     57    59    61    63    65    67    69    71    73    75

     GTCATCGGCTTCGCCATCAGCCAGCAGAAAAAGGACGTGCGGATGCTGAGATTCATCCAG
 241 ---------+---------+---------+---------+---------+---------+
     CAGTAGCCGAAGCGGTAGTCGGTCGTCTTTTTCCTGCACGCCTACGACTCTAAGTAGGTC
     V__I__G__F__A__I__S__Q__Q__K__K__D__V__R__M__L__R__F__I__Q__
     77    79    81    83    85    87    89    91    93    95

     GAAGTGAACACCACCACCAGATCCTGCGCCCTGTGGGACCTGGAAGAGGACACCGAGTAC
 301 ---------+---------+---------+---------+---------+---------+
     CTTCACTTGTGGTGGTGGTCTAGGACGCGGGACACCCTGGACCTTCTCCTGTGGCTCATG
     E__V__N__T__T__T__R__S__C__A__L__W__D__L__E__E__D__T__E__Y__
     97    99    101   103   105   107   109   111   113   115

     ATCGTGCACGTGCAGGCCATCAGCATCCAGGGCCAGAGCCCTGCCAGCGAGCCCGTGCTG
 361 ---------+---------+---------+---------+---------+---------+
     TAGCACGTGCACGTCCGGTAGTCGTAGGTCCCGGTCTCGGGACGGTCGCTCGGGCACGAC
     I__V__H__V__Q__A__I__S__I__Q__G__Q__S__P__A__S__E__P__V__L__
     117   119   121   123   125   127   129   131   133   135

     TTCAAGACCCCCAGAGAAGCCGAGAAGATGGCCTCCAAGAACAAGGACGAAGTGACCATG
 421 ---------+---------+---------+---------+---------+---------+
     AAGTTCTGGGGGTCTCTTCGGCTCTTCTACCGGAGGTTCTTGTTCCTGCTTCACTGGTAC
     F__K__T__P__R__E__A__E__K__M__A__S__K__N__K__D__E__V__T__M__
     137   139   141   143   145   147   149   151   153   155

                          PvuII                 BclI
     AAGGAAATGGGCAGAAACCAGCAGCTGAGAACCGGCGAGGTGCTGATCATCGTGGTGGTG
 481 ---------+---------+---------+---------+---------+---------+
     TTCCTTTACCCGTCTTTGGTCGTCGACTCTTGGCCGCTCCACGACTAGTAGCACCACCAC
     K__E__M__G__R__N__Q__Q__L__R__T__G__E__V__L__I__I__V__V__V__
     157   159   161   163   165   167   169   171   173   175

                                     PstI
     CTGTTCATGTGGGCTGGCGTGATCGCCCTGTTCTGCAGACAGTACGACATCATCAAGGAC
 541 ---------+---------+---------+---------+---------+---------+
     GACAAGTACACCCGACCGCACTAGCGGGACAAGACGTCTGTCATGCTGTAGTAGTTCCTG
     L__F__M__W__A__G__V__I__A__L__F__C__R__Q__Y__D__I__I__K__D__
     177   179   181   183   185   187   189   191   193   195

     AACGAGCCCAACAACAACAAAGAAAAGACCAAGAGCGCCAGCGAGACAAGCACCCCCGAG
 601 ---------+---------+---------+---------+---------+---------+
     TTGCTCGGGTTGTTGTTGTTTCTTTTCTGGTTCTCGCGGTCGCTCTGTTCGTGGGGGCTC
     N__E__P__N__N__N__K__E__K__T__K__S__A__S__E__T__S__T__P__E__
     197   199   201   203   205   207   209   211   213   215

                                               NotI
                StuI                BglII       EagI
     CACCAGGGCGGAGGCCTGCTGAGAAGCAAGATCTGATGAGCGGCCGC
 661 ---------+---------+---------+---------+-------
     GTGGTCCCGCCTCCGGACGACTCTTCGTTCTAGACTACTCGCCGGCG
     H__Q__G__G__G__L__L__R__S__K__I__
     217   219   221   223   225   227
```

2 Supplementary Figures

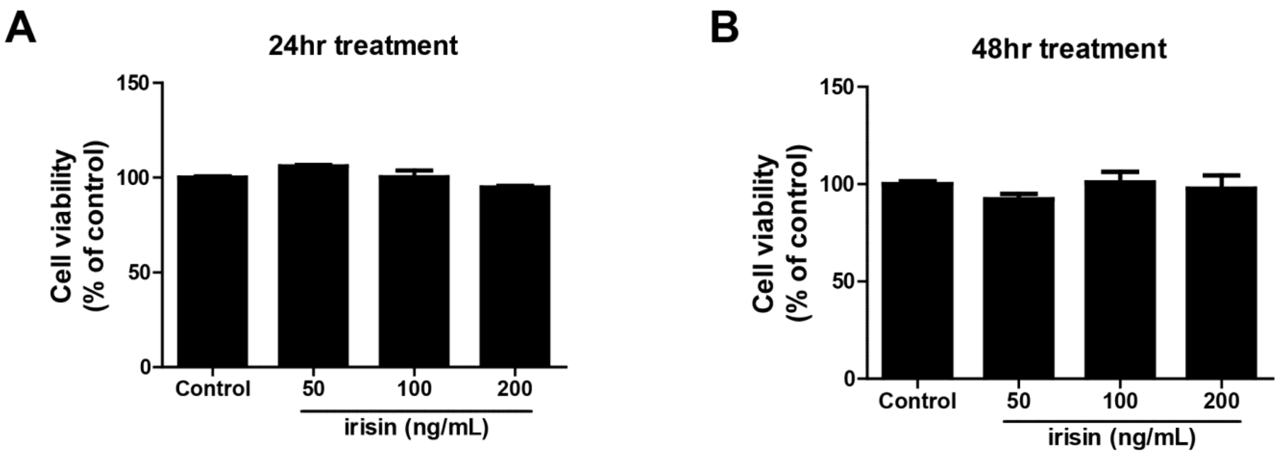

Supplementary Figure S1. The effect of recombinant irisin on cell viability (A-B) 3T3-L1 cells were treated with various concentrations of irisin for 24 and 48 hours. Cell viability was measured using MTT assay reagent.

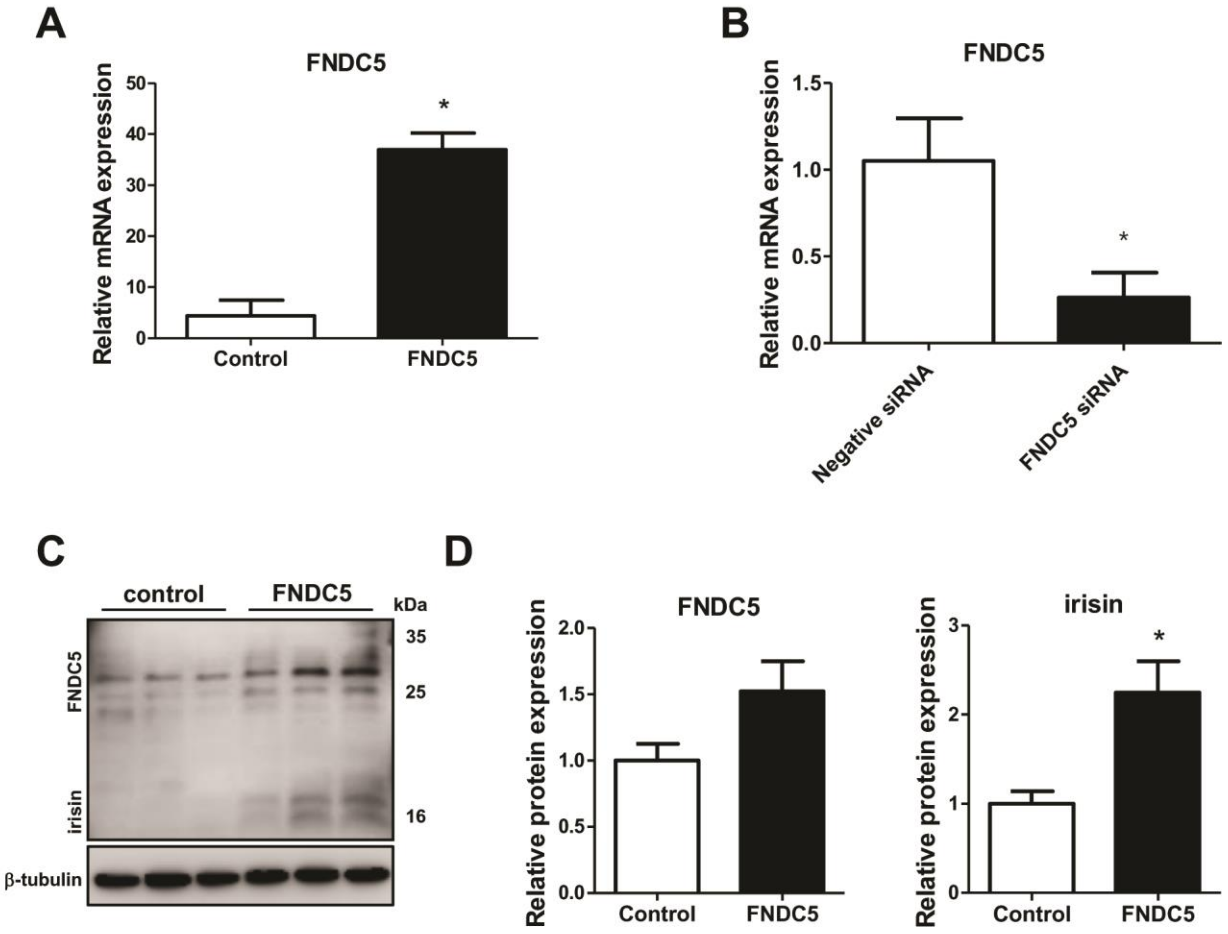

Supplementary Figure S2. The efficiency of cell transfection. (A) FNDC5 was overexpressed in 3T3-L1 preadipocytes using plasmid encoding FNDC5 gene. (B) FNDC5 was knocked down in 3T3-L1 preadipocytes using 25 nmol/L siRNA. Gene expression of FNDC5 was examined by real-time PCR and normalized to 18s. (C, D) Western blot analysis of FNDC5 and irisin protein expression in 3T3-L1 preadipocytes overexpressed with FNDC5. Each target has been normalized with β-tubulin as an internal control. *$P < 0.05$ compared with control.
